# Detection of biogenic magnetic nanoparticles in rapidly dividing tumor cells by the nonlinear magnetization method

**DOI:** 10.3389/fbioe.2025.1680057

**Published:** 2025-10-27

**Authors:** Marina V. Milovanova, Anna N. Gabashvili, Elizaveta N. Mochalova, Ekaterina O. Gurtovaya, Irina E. Egorova, Anastasiia A. Dresviannikova, Olga Yu. Griaznova, Petr I. Nikitin

**Affiliations:** ^1^ Prokhorov General Physics Institute of the Russian Academy of Sciences, Moscow, Russia; ^2^ Central Research Institute of Epidemiology of the Federal Service for Surveillance on Consumer Rights Protection and Human Wellbeing, Moscow, Russia; ^3^ Moscow Center for Advanced Studies, Moscow, Russia; ^4^ Sirius University of Science and Technology, Sirius, Krasnodar region, Russia; ^5^ Biotechnological Faculty, Lomonosov Moscow State University, Moscow, Russia; ^6^ Shemyakin-Ovchinnikov Institute of Bioorganic Chemistry of the Russian Academy of Sciences, Moscow, Russia

**Keywords:** magnetic particle quantification, magnetic nanoparticles, multimodal detection, protein-based nanomaterials, optical imaging, biogenic nanoparticles, encapsulins, *in vitro* and *in vivo* monitoring

## Abstract

**Introduction:**

Genetically encoded nanoplatforms–bacterial nanocompartments (encapsulins) have demonstrated a remarkable capacity for innovation in the fields of biomedicine and biotechnology. These platforms have found novel applications in a variety of approaches, including magnetic resonance imaging (MRI), transmission electron microscopy (TEM), and high-resolution microscopy, among others. Particular attention has been given to the encapsulin system of the bacterium Quasibacillus thermotolerans (Qt). Divalent iron has been found to sequester within Qt shells, resulting in the formation of biogenic magnetic ferric oxide nanoparticles (MNPs) with T2 contrast properties. Recent studies have led to the successful obtaining of mammalian cells that stably express Qt genes and are capable of producing MNPs. These cells can be detected *in vitro* and *in vivo* using both MRI and the nonlinear magnetization method (magnetic particle quantification (MPQ) method). The objective of this study was to investigate the advantages and limitations of labeling mammalian cells with the Qt encapsulins.

**Methods:**

A rat C6 glioma cell line was engineered to express a red fluorescent protein (RFP) as an optical tag and a Qt nanocompartment as a magnetic tag by lentiviral transduction. The generated C6-RFP-Qt cells were characterized by inductively coupled plasma mass spectrometry (ICP-MS) and Perls staining as well as using the MPQ technique, fluorescent microscopy, and optical tomography. The in vivo study was conducted using severe combined immunodeficient (SCID) mice.

**Results:**

A prominent in vivo model of glioblastoma multiforme has undergone substantial enhancement. The magnetic signal retention time in C6-RFP-Qt cells was first estimated by the MPQ technique.

**Discussion:**

The findings indicated the potential for real-time monitoring of magnetic signal amplitude during cell proliferation process utilizing the MPQ method. The approach employed constitutes a simple yet more sensitive alternative to conventional methods for studying MNPs.

## 1 Introduction

Glioblastoma multiforme (GBM) is a highly invasive and aggressive low-grade glioma, characterized by rapid tumor cell proliferation and a high rate of recurrence following surgical resection (Hosseininia et al., 2024). The presence of several features, including the high heterogeneity of GBM, its rapid progression, radio- and chemoresistance, and numerous other properties, poses significant challenges to the development of both GBM therapeutics and diagnostics. The primary therapeutic interventions for this neoplasm are centered on personalized therapy regimens, with the objective of enhancing the efficacy of drug delivery across the blood-brain barrier (Burko et al., 2023). A wide variety of mouse and rat glioblastoma models are utilized to study glioma growth and development, as well as tumor response to therapy. These models encompass syngeneic and xenogeneic models (Barth and Kaur, 2009; Doblas et al., 2010; Doblas et al., 2012; Huszthy et al., 2012; Sahu et al., 2022), with rat glioma C6 being the gold standard due to its ability to mimic the characteristics of human GBMs (Lim et al., 2025).

The utilization of both native and transgenic cells in the modeling of glioblastoma is a well-established practice (Cai et al., 2023; Clements et al., 2024). Nevertheless, the tracking of fluorescently labeled glioma cells *in vivo* poses significant challenges due to the low penetration depth of the signal. To overcome this obstacle, various magnetic labels (Marie et al., 2015), and radiotracers (Pan et al., 2024) are used for direct glioma cells labeling. As was mentioned, in the case of rapidly dividing cells, direct labeling is not the most effective approach, and this applies to both magnetic and optical tags. Another option to consider is the genetic modification of glioblastoma cells with MRI (magnetic resonance imaging) reporter genes such as ferritin (Cohen et al., 2005) or lysine rich–protein (Gilad et al., 2007). At last, there are approaches that involve several different imaging techniques at once. For instance, a combination of 18F-FET (18F-fluoroethyltyrosine) PET (positron emission tomography) and CEST (chemical exchange saturation transfer) MRI was performed for glioma characterization in rats (Jackson et al., 2022). Another article describes the possibility of studying the progression of glioblastoma in mice using a combination of MRI and two-photon microscopy (Zomer et al., 2022). Furthermore, the study on rat L9 and C6 glioma cell lines demonstrated the feasibility of employing three distinct methods–PET, MRI, and optical imaging–in a concurrent manner (Scarpelli et al., 2020).

As previously mentioned, the rapid proliferation of malignant glioma cells considerably hinders the utilization of exogenous nanoparticles for long-term monitoring of these cells. As stated in previous literature, there are works that claim an unrealistically high retention time of the magnetic signal in tumors modeled by C6 cells transplantation, which does not correspond to actual tumor growth rates. For instance, in one of the analyzed works, the authors employed poly-L-lysine-conjugated superparamagnetic iron oxide particles (PLL-SPIO) for *in vitro* labeling of C6 cells with their subsequent transplantation to obtain orthotopic tumors in rats. The study asserts that the methodology employed enables the monitoring of tumor cell migration for a period of 20 days subsequent to transplantation. As demonstrated in the T2- and T2*-weighted images, the presence of MNPs is evident in the form of hypointense areas. However, these areas are predominantly located in the central regions of the tumor stroma. These results are consistent with histological data showing isolated Perls-stained cells within the tumor mass (Zhang et al., 2011). Additionally, the MR signal’s localization to tumor cells cannot be definitively attributed, as there is a significant possibility that the MNPs were captured by brain microglial cells. In more recent articles devoted to *in vivo* studies of gliomas, the imaging strategy has evolved toward the utilization of contrast agents that selectively accumulate in the tumor after systemic administration (Tao et al., 2024). Nevertheless, this modification in methodology is inadequate for resolving the issue of signal intensity decline during cell division. In another study, the authors evaluated the potential for the retention of the iron nanoparticle label, and the resulting MRI signal, to serve as a marker for slowly dividing cancer cells. The results were obtained by using micron-sized superparamagnetic iron oxide (MPIO) nanoparticles to study GFP-expressing MDA-MB-231 breast cancer cells growth and metastasis. The presence of small focal regions of signal loss was detected in images of the axillary and brachial nodes at day 14 after the injection of MDA-MB-231 cells (Economopoulos et al., 2013).

The issue of the limited signal retention time in rapidly dividing cells has emerged as a significant constraint in the utilization of diverse nanoplatforms for translational research. These limitations are associated not only with the complexity of tumor imaging but also with the use of nanoagents in various therapeutic approaches. In the domain of photothermal therapy, nanoparticles have been demonstrated to target and ablate cancer cells through the induction of hyperthermia (Rivera-Rodriguez et al., 2018; Chang et al., 2025). Similarly, in photoimmunotherapy, MNPs have been employed to enhance the delivery of antibody-photo-absorber-conjugates to specific cancer cell antigens (Tao et al., 2014; Wang et al., 2024). In the context of rapidly proliferating tumor cells, this may result in a therapeutic effect that is diminished in magnitude or less well-controlled in terms of its spatial distribution. Furthermore, the absence of a persistent magnetic signal complicates the precise calculation of exposure time and intensity of magnetic fields necessary for achieving therapeutic outcomes, potentially resulting in incomplete tumor ablation or damage to surrounding healthy tissue. In the context of utilizing MNPs as drug carriers, their rapid clearance or brief signal duration can result in diminished drug release at the tumor site, thereby decreasing the drug’s efficacy (Gawali et al., 2019; Chatterjee et al., 2025).

In view of the aforementioned findings, the objective of this study is to ascertain the precise temporal parameters of magnetic signal retention in glioma cells during the proliferation process. We generated a C6-RFP-Qt cell line that contains, in addition to RFP (red fluorescent protein), *Quasibacillus thermotolerans* (Qt) iron-sequestering nanocompartments that surpass all existing proteins in terms of iron storing capacity. Bacterial nanocompartments are structurally similar to viral capsids and consist of a shell and a cargo protein inside. The shell is formed by self-assembly from identical protein subunits. In the case of Qt nanocompartments, at the points where the subunits join, pores are formed by negatively charged amino acid residues. This property allows the transport of positively charged low-molecular weight compounds into the shells (Giessen et al., 2019). In the present study Mohr’s salt (ammonium iron (II) sulfate, FAS) is used as such a compound. After entering the encapsulin shell the divalent iron in Mohr’s salt is oxidized to trivalent iron by the action of the ferritin-like cargo protein. As a result, nanoparticles with magnetic properties are formed inside the shells (Gabashvili et al., 2020; Giessen, 2022).

The MPQ technique was utilized as the signal detection method, as it is the most sensitive approach to detect nonlinear magnetic materials. Its superiority to conventional methods, such as MRI and the superconductor quantum interference device (SQUID) magnetometry, has been demonstrated (Nikitin et al., 2008; Zelepukin et al., 2021).

In this study, we have improved one of the most prevalent *in vivo* models of GBM. An examination of the obtained C6-RFP-Qt cells in severe combined immunodeficient (SCID) mice has been conducted, thereby substantiating the capacity for the formation of subcutaneous xenograft tumors subsequent to the transplantation. Utilizing the Prussian blue staining reaction, we have dynamically assessed the accumulation of trivalent iron in C6-RFP-Qt cells following incubation with FAS and for a period of 8 days of cell incubation in a FAS-free medium. It has been determined that the distinctive blue coloration resulting from the presence of ferric ferrocyanide can be discerned in C6-RFP-Qt cells up to the fifth day of cultivation in a FAS-free medium. The findings from this study are supported by the data obtained by the MPQ method. By the fifth day of cell culturing in FAS-free medium, the magnitude of the normalized magnetic signal in C6-RFP-Qt cell samples took a value that was not statistically significantly different from the value in the control samples. While the nanoplatform used has previously demonstrated its efficacy in long-term, non-invasive stem cell tracking, it can be deduced that the utilization of such cell labeling tactics is suboptimal for the study of rapidly growing solid tumors.

## 2 Materials and methods

### 2.1 Cell lines and culture

C6 rat glioma cells were cultivated in Dulbecco modified Eagle medium (DMEM, Gibco, Waltham, MA, USA) supplemented with 2 mM L-glutamine (Gibco, Waltham, MA, USA), antibiotics (100 U/mL penicillin, 0.1 mg/mL streptomycin, Gibco, Waltham, MA, USA) and 5% fetal bovine serum (FBS, Cytiva, Marlborough, MA, USA) under standard conditions at 37  C and 5% CO_2_, in T75 culture flasks (NEST Biotechnology, Wuxi, China). At 70%–80% monolayer confluency, adhesive cells were harvested following trypsinization (0.25% trypsin-EDTA solution, Gibco, Waltham, MA, USA) and cultivated in a ratio of 1:4 or 1:10. 293T cells were cultured in T25 cell culture flasks (NEST Biotechnology, Wuxi, China) in DMEM supplemented with 2 mM L-glutamine, antibiotics (100 U/mL penicillin, 0.1 mg/mL streptomycin, all purchased from Gibco, Waltham, MA, USA) and 10% FBS (Cytiva, Marlborough, MA, USA) under standard conditions.

### 2.2 Assembly of lentiviral vectors and lentiviral transduction

The construction of lentiviral vectors was carried out using the 293T packaging cell line and Lipofectamine 3000 Transfection kit (Thermo Fisher Scientific, Waltham, MA, USA) in accordance with the manufacturer’s protocol. Тo assemble lentiviral vectors, packaging plasmids (pRSV-Rev, pMDLg/pRRE, and pCMV-VSV-G, Addgene, Watertown, MA, USA) and transport plasmids (pLCMV QtEncFLAG-QtIMEF/pLCMV mZip14/pLCMV-tagRFP-puro, Supplementary Figure S1) were mixed with P3000 reagent in Opti-MEM medium. Diluted plasmid DNA was then added to diluted Lipofectamin3000 reagent in OptiMEM medium (in a 1:1 ratio), and after 20 min of incubation, the resulting DNA-lipid complexes were added to the cells in 6-well plates. Twenty-four hours after the transfection, the medium was aspirated and substituted with a complete DMEM growth medium. Lentiviral stocks were harvested 48 h and 72 h after transfection and filtered through a 0.45 μm syringe filter. The transduction of rat C6 glioma cells with lentiviral vectors was conducted in accordance with the standard protocol in DMEM, with the addition of 10% heat-inactivated FBS and 10 μg/mL polybrene (Merck, Darmstadt, Germany). Following a 48-hour period of transduction, selection was initiated using puromycin (Thermo Fisher Scientific, Waltham, MA, USA) at a concentration of 1 μg/mL; the medium with puromycin was changed to a new one every other day. The selection was continued for 10 days. The resulting cell line was designated C6-RFP-Qt.

### 2.3 Reverse transcription polymerase chain reaction (RT-PCR)

Transduced cells cultivated in T25 flasks, achieving up to 90% monolayer density. Total RNA was isolated using a commercial RNA extraction reagent (Evrogen, Moscow, Russia) according to a standard protocol. Spectrophotometry was employed to evaluate the concentration and quality of RNA. cDNA was synthesized by SuperScript III Reverse Transcriptase (Thermo Fisher Scientific, Waltham, MA, USA), oligo-DT and random primers. Subsequently, the resulting cDNA samples and a negative control (RNA sample without the addition of reverse transcriptase during cDNA synthesis) were used for classical PCR with Taq polymerase (Fermentas, Waltham, MA, USA) and the appropriate buffer. The separation of the PCR products was conducted through the use of electrophoresis in 1% agarose gel.

### 2.4 Western blot analysis

Western blot analysis was conducted in accordance with a standard protocol, albeit with minor modifications. Prior to analysis, C6-RFP-Qt cells were subjected to lysis with RIPA (radioimmunoprecipitation assay) buffer, after which 5X sample buffer was added to 10 μL of the cell lysate. The samples were heated at 95 °C, followed by cooling on ice, and subsequent application to a gel. The gel electrophoresis was conducted at 80 V for a duration of 25 min followed by a subsequent application of 100 V for a period of 1.5 h. The gel was then placed in the transfer buffer, and the nitrocellulose membrane was activated and placed over the gel. The transfer was performed for 1 h at 100 V. The membrane was than washed three times from the residual transfer buffer in PBS containing 0.1% Tween 20 (PBST). In order to block nonspecific protein binding, the membrane was incubated for 2 h in a solution of 5% skimmed milk in PBST. Afterward, the membrane was incubated with Alexa Fluor 488 anti-DYKDDDDK Tag Antibody (BioLegend, San Diego, CA, USA, 1:1000) for 2 h, followed by three washing steps. The membrane then was exposed with secondary goat anti-mouse alkaline horseradish peroxidase-conjugated IgG antibodies (Santa Cruz Biotechnology, Dallas, TX, USA, 1:1000). PageRuler (Thermo Fisher Scientific, Waltham, MA, USA) was utilized as a marker of length. Clarity Max Western ECL Substrate kit (BioRad, Hercules, CA, USA) was employed to reveal the result. The results were captured using the ChemidocMP imaging system (BioRad, Hercules, CA, USA).

### 2.5 Fluorescent microscopy

Fluorescent micrographs of C6-RFP/C6-RFP-Qt cells (TagRFP excitation/emission maxima at 555 and 584 nm, respectively) were captured with the FLoid Cell Imaging Station (Thermo Fisher Scientific, Waltham, MA, USA).

### 2.6 Cytotoxicity study of ferrous ammonium sulphate (FAS)

The impact of FAS on the viability of C6-RFP and C6-RFP-Qt cells was evaluated via the resazurin assay, according to the manufacturer’s instructions. C6-RFP and C6-RFP-Qt cells were seeded into the wells of a 96-well opaque culture plate at a density of 8 × 10^3^ cells per well in 100 μL of growth medium. Following 24 h of culture, FAS (Sigma-Aldrich, St. Louis, MI, USA) was added to the cells at varying concentrations (0, 0.5, 1, 2, 4, and 10 mM). After another 24 h of incubation, the cells were washed with PBS, and fresh growth medium containing 50 μM resazurin sodium salt (Santa Cruz Biotechnology, USA) was added to each well. Cells that had not been treated with FAS served as the positive control. The cells were incubated with resazurin for 4 h at 37 °C and 5% CO_2_ within a humidified atmosphere. The analysis was performed in triplicate, and the intensity of the fluorescent signal of resorufin (λ_Ex_ = 560 nm/λ_Em_ = 590 nm) was quantified using a Feyond A400 (Hangzhou Allsheng Instruments, China) microplate reader (λ_Ex_ = 523 nm; λ_Em_ = 564 nm).

Cell viability was calculated using the following formula:

$$\text{Cell viability }\left(\%\right)=\left(\text{As}-\text{Ab}\right)/\left(\text{Ac}-\text{Ab}\right)\times 100,$$

where As represents the average fluorescence intensity in the experimental wells, Ab denotes the average fluorescence intensity in the blank (wells containing medium without cells), and Ac refers to the average fluorescence intensity in the wells of the positive control.

### 2.7 Inductively coupled plasma mass spectrometry (ICP-MS)

C6-RFP-Qt and C6-RFP (control) cells were cultured in 24-well plates at a density of 2 × 10^5^ cells per well. The evaluation of intracellular iron accumulation was conducted by adding FAS to the growth medium at concentrations of 0.5 mM, 1 mM, 2 mM, and 4 mM, followed by a 24-h incubation period. Thereafter, the cells were thoroughly washed with PBS, detached from plastic via trypsin solution, and pelleted by centrifugation before counting. Each cell sample (6 × 10^5^ cells) was then dissolved in 100 µL of 68% nitric acid (Chimmed, Moscow, Russia) and incubated for 2 h at 60 °C. The concentration of iron was subsequently determined utilizing a NexION 2000 ICP mass spectrometer (PerkinElmer, Waltham, MA, USA).

### 2.8 Magnetic-activated cell sorting (MACS)

MACS was performed utilizing a commercial MS Column (Miltenyi Biotec, Bergisch Gladbach, Germany) alongside a MiniMACS magnetic separator (Miltenyi Biotec, Bergisch Gladbach, Germany). C6-RFP-Qt cells were cultured in a T75 cell culture flask until 90% confluency was attained. Subsequently, the cells were exposed to 4 mM FAS for a period of 24 h. The cells were subsequently detached from the flask through trypsinization and resuspended in 2 mL of PBS buffer, which was supplemented with 2% FBS. The magnetic column was placed within the magnetic field generated by the separator. Thereafter, 1 mL of PBS +2% FBS buffer was passed through the column to equilibrate it. With the column prepared, the sorting process commenced. The cells (up to 1 × 10^7^ C6-RFP-Qt in 500 µL of PBS +2% FBS buffer) were passed through the column, with the cells that flowed freely being collected in a 15 mL tube. The column was then washed three times with a PBS +2% FBS solution. Subsequent to the completion of the washing process, the column was detached from the magnetic field of the separator. Thereafter, the cells that had been retained within the column were eluted in 1 mL of PBS +2% FBS into a separate tube using a plunger. The sorted cells (approximately 3% of the initial cell count) were subsequently seeded into the wells of a 96-well plate for further cultivation.

### 2.9 Prussian blue staining

The staining of the cells was carried out in accordance with the manufacturer’s guidelines of the Prussian blue iron Staining Kit (Biovitrum, Moscow, Russia). Images were taken using a CX41 FLLED light microscope (Ningbo Sunny Instruments, Yuyao City, China) and analyzed by ToupView v. 4.1 Software (ToupTek Photonics, Hangzhou, China).

### 2.10 Animal study

All procedures were approved by the Institutional Animal Care and Use Committee of the Shemyakin-Ovchinnikov Institute of Bioorganic Chemistry Russian Academy of Sciences according to protocol #375/2023 (20 September 2023–19 September 2026). Male SCID mice with a body weight of 24–26 g were utilized in the study. The mice were housed in a temperature-controlled facility under a 12 h photoperiod, no more than ten per cage. The mice were given food and water *ad libitum*. The animals were anesthetized by intraperitoneal injection of a Zoletil 100/Xyla combination at the dose of 40/1.5 mg/kg. Mice were subcutaneously inoculated with 5 × 10^6^ C6-RFP/C6-RFP-Qt cells in 100 μL of SFM (serum-free media) into the right flank to create tumor xenografts.

### 2.11 Fluorescent optical tomography

Fluorescent RFP-channel images of the animals/extracted organs were obtained using a LumoTrace FLUO tomograph (Abisense, Sirius, Russia) using 550 nm diodes and a 600+ nm filter with an exposure time of 3000 ms. Images in the bright field mode were recorded with an exposure time of 100 ms. For the mice injected with C6-RFP-Qt cells, the fluorescence signal mask was intensity-thresholded based on the autofluorescence levels measured in non-injected control mice. These control animals, which were imaged alongside each mouse of interest in the original images, provided the baseline for defining the specific signal.

### 2.12 Magnetic particle quantification (MPQ) analysis of cells

The analysis was conducted using the updated MPQ device employing the method of nonlinear magnetization. This methodology is predicated on the application of an alternating magnetic field generated by two distinct frequencies, *f*1 and *f*2. A sample is positioned within this generated field, and due to the properties of magnetic nanoparticles as nonlinear magnetic materials, a signal is induced at combinatorial frequencies, which represent a linear combination of *f*1 and *f*2 (Nikitin et al., 2009; Nikitin et al., 2018).

## 3 Results and discussion

### 3.1 C6 glioma cells modified with Qt nanocompartment genes are capable of producing MNPs upon addition of FAS to the growth medium

**FIGURE 1:**
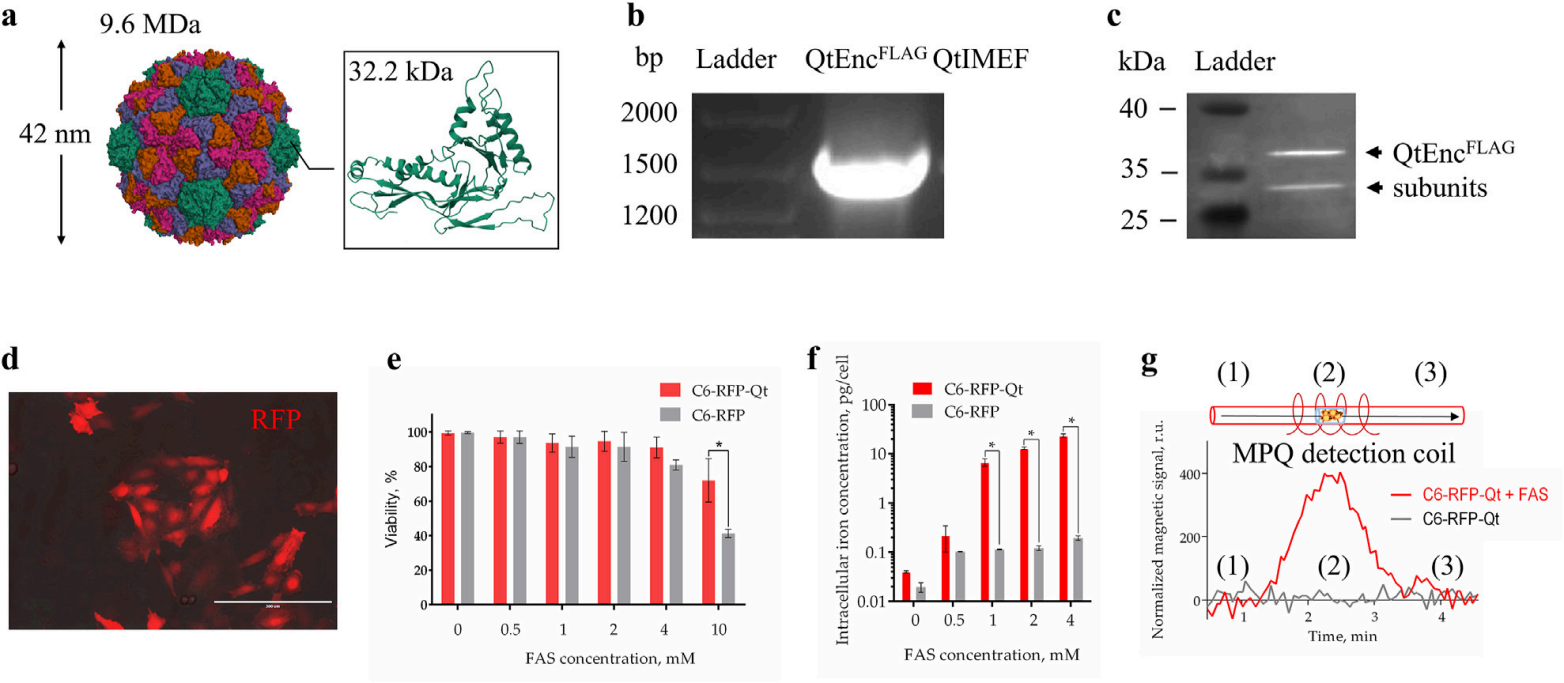
Characterization of dual-labeled C6 glioma cells. **(a)** Overall structure of full-assembled icosahedral Qt encapsulin shell and structure of a single QtEnc subunit (PDB entry: 6NJ8); **(b)** RT-PCR analysis of C6-RFP-Qt cells, the sequence of interest was identified by its length; **(c)** WB analysis of FLAG-tagged QtEnc proteins (arrows indicate bands with molecular weight of ∼35 kDa); **(d)** fluorescent photomicrograph of C6-RFP-Qt cells, red fluorescent signal–RFP, scale bar – 100 μm; **(e)** resazurin viability assay of C6-RFP-Qt and C6-RFP cells in the presence of different concentrations of FAS in cell culture medium; **(f)** intracellular iron content in 6 × 10^5^ of C6-RFP-Qt and C6-RFP cells measured by ICP-MS (inductively coupled plasma mass spectrometry); **(g)** magnetic signals in samples of C6-RFP-Qt before (control) and after incubation with 4 mM Mohr’s salt analyzed via the MPQ approach, (1) and (3) samples are outside the coil, (2) samples are inside the MPQ detection coil. All data are shown as mean ± SD of tree independent experiments, * indicate *p* < 0.05.

A salient feature of MNPs synthesized within encapsulin shells pertains to the genetic basis of their synthesis. In contrast to MNPs produced through chemical methods, the dimensions of MNPs biosynthesized into nanocompartments are strictly constrained by the dimensions of the protein shells. The size of the genetically encoded shell of Qt encapsulins is 42 nm (Figure 1a), and the size of MNPs formed inside such shells is about 25 nm (Efremova et al., 2021; Gabashvili et al., 2023).

The employment of lentiviral vector facilitated the establishment of a stable cell line in which the insert of interest, comprising the shell and cargo proteins of Qt encapsulins, is maintained during the process of cell proliferation (Figure 1b). To enhance the uptake of Fe^2+^ into cells, mZip14 divalent metal transporter genes were also introduced as an auxiliary element. The FLAG-tagged QtEnc subunits were identified through the implementation of an immunoblotting technique. Two bands with a molecular weight of approximately 35 kDa were observed in the Western blot analysis (Figure 1c). This phenomenon may be due to the fact that encapsulins are able to dynamically disassemble/reassemble under different conditions. For example, encapsulins are stable between pH 4.5 and 8.5, but can reversibly disassociate under strong acidic and alkaline conditions (Jones et al., 2021). It is known that *Thermotoga maritima* encapsulins are resistant to thermal disassembly and remain stable even after heating to 90 °C. It was also found that Qt encapsulins shells undergo structural change from 40 °C but remain assembled until 80 *°*C (Boyton et al., 2022). It has been demonstrated that PEGylated hollow engineered encapsulin nanoparticles from *Rhodococcus erythropolis* N771 (*R. erythropolis* N771) can effectively disassemble and reassemble following PEGylation. The engineered PEGylated nanocompartments were disassembled into identical encapsulin subunits by the addition of guanidinium hydrochloride (GdnHCl), and successfully reassembled by dialysis (Sonotaki et al., 2017). One of the studies showed that zeolites (crystalline aluminosilicate materials with strictly ordered porous structures) can facilitate a method for preparing successfully reassembled PEGylated nanocompartments from *R. erythropolis* N771 without the use of protein denaturants throughout the proteins’ reassembly process (Sonotaki et al., 2019). Furthermore, a recent cryo-electron tomography study of *Mycobacterium marinum* (*M. marinum*) nanocompartments revealed encapsulins with heterogeneous cargoes and partially assembled (or disassembled) encapsulin shells. A thorough examination of the occupancy of dimers, trimers, and pentamers in *M. marinum* nanocompartments shell structures indicates that encapsulins are predominantly assembled from dimers, as opposed to trimers or pentamers (Berger et al., 2025). Earlier literature has also documented the observation of partially assembled encapsulins with cargo inside (Snijder et al., 2016).

The subsequent stage of the experiment involved the characterization of the optic label in the resulting cell line. The presence of a bright fluorescent signal of RFP was detected through the use of fluorescent microscopy (Figure 1d). The control cell line, which contained RFP exclusively, had previously been obtained and studied by us. It has been demonstrated that C6-RFP cells, when engrafted into the brain of immunocompetent rats, exhibited an infiltrating growth pattern and perivascular invasion, characteristics that are hallmarks of GBM.

In order to enhance the capacity for magnetic signal detection in cells MACS (magnetic activated cell sorting) was employed to separate a subpopulation of C6-RFP-Qt that exhibited the highest density of nanocompartments per cell. A subsequent comparison was made of the viability of the obtained C6-RFP-Qt cells and control cells in the presence of different concentrations of Mohr’s salt. It is known that when exposed to reactive oxygen species, ferrous iron undergoes oxidation to ferric iron by the Fenton reaction, resulting in the formation of a hydroxyl radical as a by-product (Imlay et al., 1988; Andrews, 1998). Ferritins have been observed to protect cells against the deleterious effects of this by-product. It has been demonstrated that nanocompartments can similarly protect a cell from oxidative stress. For instance, in one study, the survival of a *Myxococcus xanthus* mutant strain lacking the sequence encoding the encapsulin shell under conditions of oxidative stress induced by hydrogen peroxide was evaluated. The results demonstrated that the mutant strain exhibited a significant increase in sensitivity to hydrogen peroxide compared to the wild-type strain (McHugh et al., 2014). Encapsulin-associated ferritin-like proteins (FLPs) have also been observed to exhibit ferroxidase activity, with the encapsulin shell functioning as a storage compartment for mineralized iron. The FLP-type IMEF cargo proteins from Qt encapsulins assemble into dimers of four-helix bundles and form a dinuclear iron ferroxidase center, containing the iron binding sites at the dimer subunit interface (Giessen et al., 2019). The hypothesis is that the presence of these sites enables IMEF to catalyze Fe^2+^ oxidation to Fe^3+^ through an Fe^4+^ intermediate and the release of water. The encapsulin shell provides a substantial storage compartment for the soluble and bioavailable dynamic storage of iron—larger than ferritin or bacterioferritin cages—and establishes a selectively permeable diffusion barrier for Fe^2+^ influx. This results in the control of the internal ferrous iron concentration and the prevention of uncontrolled iron precipitation by encapsulated FLPs (Fazal and Giessen, 2025). Therefore, in C6-RFP-Qt cells the concentration of labile cytoplasmic iron will decrease due to accumulation into encapsulins, resulting in improved vitality in the presence of FAS (Figure 1e).

Subsequently, an evaluation was conducted to quantify the intracellular iron content following the incubation of C6-RFP-Qt and C6-RFP cells with Mohr’s salt in four distinct concentrations (0.5, 1, 2, and 4 mM). The findings indicated that the accumulation of iron within C6-RFP-Qt cells exhibited a dose-dependent manner, and that the iron concentration in cells containing encapsulins was statistically significantly higher in comparison to the control group (Figure 1f).

Finally, the magnetic signals of C6-RFP-Qt cells were measured before and after incubation with FAS using the MPQ method. As demonstrated in Figure 1g, the magnitude of the normalized magnetic signal in a sample of 5×10^5^ C6-RFP-Qt cells after incubation with 4 mM Mohr’s salt was significantly higher than in the control sample, by more than an order of magnitude.

**FIGURE 2:**
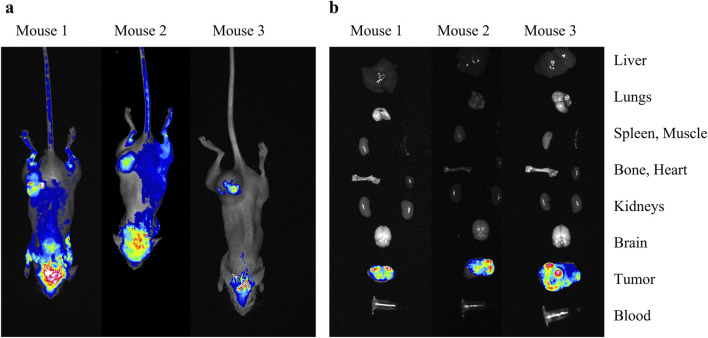
*In vivo* **(a)** and *ex vivo* **(b)** study of the heterotopic tumors that developed after subcutaneous transplantation of C6-RFP-Qt cells. Merged brightfield and RFP-channel images of mice/isolated organs.

The generated cells were next transplanted subcutaneously into SCID mice. The cells engrafted successfully and subsequently formed heterotopic tumors. To assess the stability of the fluorescent label, *in vivo* (Figure 2a) and *ex vivo* optical tomography imaging was performed. For the *ex vivo* study, we conducted optical tomography of tumors and key organs (liver, lungs, spleen, heart, kidneys, brain) as well as muscle, bone and blood (Figure 2b). The RFP fluorescence signal was found to be present in C6-RFP-Qt cells throughout the development of the tumor. A schematic illustrating the comprehensive workflow, from the generation of a transgenic cell line to animal studies, is presented in Supplementary Figure S2.

The employment of a subcutaneous xenograft model has facilitated the successful demonstration of the proof-of-concept for fluorescent signal tracking in C6-RFP-Qt cells. The utilization of subcutaneous xenografts is predicated on the capacity of this method to facilitate noninvasive visualization of the fluorescent label. However, it must be acknowledged that this approach is not without its inherent limitations. These include the absence of the brain-specific microenvironment, encompassing its distinctive vascular architecture, immune context, and the blood-brain barrier, which plays a critical role in assessing therapy response (Huszthy et al., 2012; Sahu et al., 2022). The utilization of an orthotopic glioma facilitates the assessment of tumor dynamics under conditions that closely resemble the actual pathophysiology of glioblastoma. The intracranial implantation of C6-RFP-Qt cells is imperative for the investigation of tumor-microglia interactions and the characteristics of invasive growth within the brain parenchyma. However, for *in vivo* fluorescence signal detection, it is necessary to replace the fluorescent label with a near-infrared fluorophore to enhance signal penetration depth.

The subsequent objective was to determine how long the quantity of iron MNPs in C6-RFP-Qt cells remains sufficient for the detection of the magnetic signal. As previously mentioned, the cargo protein with ferroxidase activity oxidizes divalent iron inside the nanocompartment shells to trivalent iron. Prussian blue (Perls) staining is a rapid and straightforward method for estimating the trivalent iron content in cells. Perls staining is a technique that utilizes a blue stain to identify the presence of ferric iron within a cell. Ferric iron reacts with soluble potassium ferrocyanide in the dye to form an insoluble pigment: hydrated ferric ferrocyanide, which is blue or violet. The method is widely used for diagnosis and research purposes both *in vitro* and *in vivo*. Prussian blue staining is employed to identify “non-heme” iron in biological samples. It is important to note that this procedure does not stain iron that is bound to porphyrin, forming heme, such as hemoglobin (Sonoda et al., 2025).

### 3.2 In the absence of a source of ferrous iron, the magnetic signal in rapidly dividing cells persists for 5 days

**FIGURE 3:**
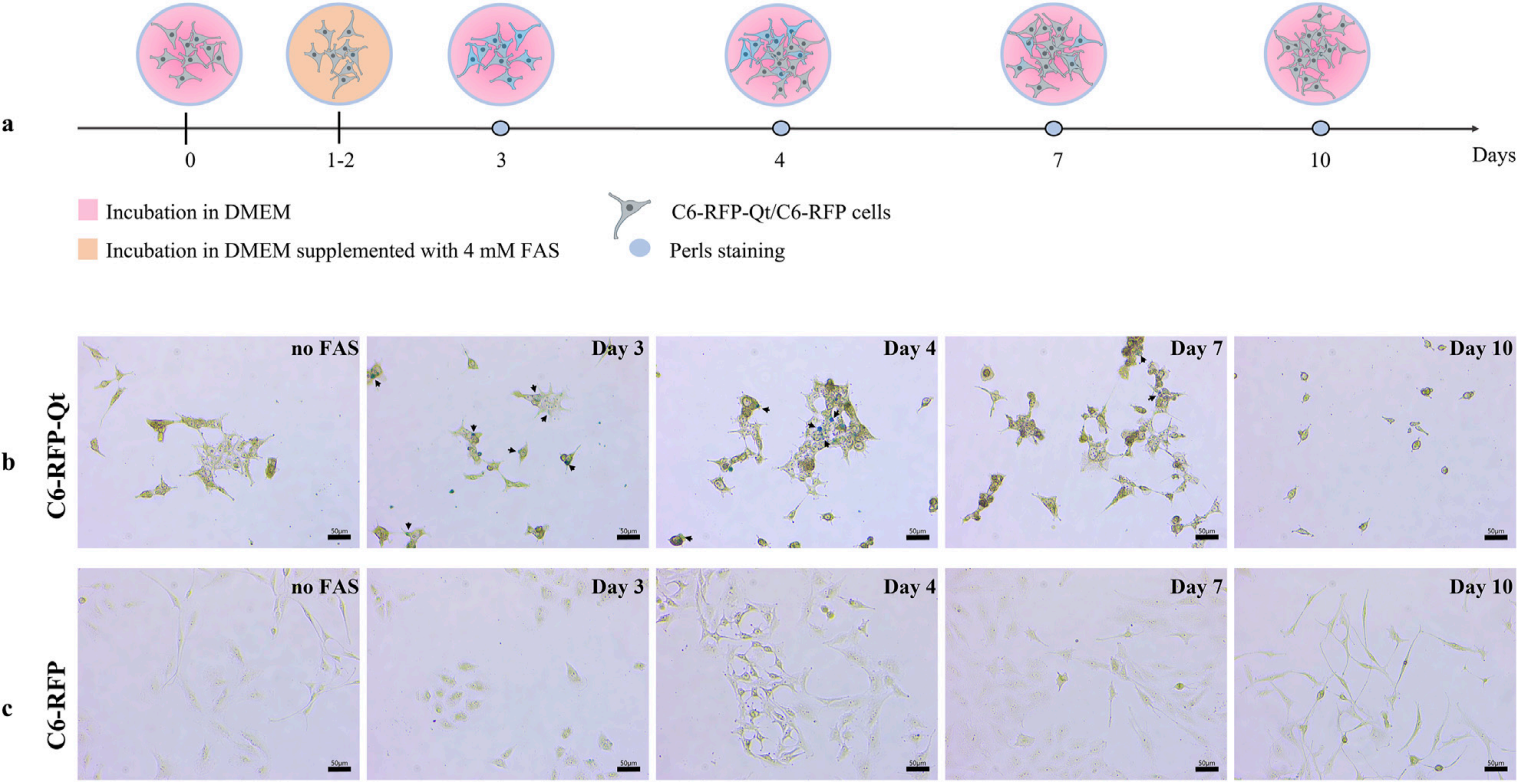
Comparative assessment of ferric iron accumulation in C6-RFP-Qt and C6-RFP cells by Prussian blue staining. Experimental design **(a)**; Perls stained C6-RFP-Qt **(b)** and C6-RFP **(c)** cells. Black arrows indicate ferric ferrocyanide in C6-RFP-Qt cells. Light microscopy, CX41 FLLED microscope, scale bars are 50 μm.

Initially, C6-RFP and C6-RFP-Qt cells were seeded on Petri dishes on day 0. On the next day (day 1), 4 mM FAS solution was added to the cells within the growth medium, and the cells were cultivated for a period of 2 days to facilitate the formation of MNPs (days 1–2). The growth medium was then substituted with a fresh, FAS-free medium, and Prussian blue staining was conducted at various time points (days 3, 4, 7, and 10, Figure 3a). The results of the study demonstrated that the light blue coloration in C6-RFP-Qt cells (Figure 3b) indicating the presence of ferric ferrocyanide in cells persisted until day 7 of the experiment. Conversely, no such coloration was observed in C6-RFP cells (Figure 3c) at any time point.

It is noteworthy that the decrease in staining intensity observed in the Prussian Blue reaction is indicative of cell proliferation rather than MNPs degradation within the nanocompartment shells. This assertion is supported by data obtained in a preliminary study conducted on a primary culture of human mesenchymal stem/stromal cells (MSCs), whose proliferation rate is significantly lower than that of glioma cells. These cells were transplanted into the brains of immunocompetent rats, and it was demonstrated that the MR signal remained stable for a period of at least 7 days following transplantation (Fedotov et al., 2021). The genetic sequence encoding the Qt iron storage system was introduced into C6 cells using a lentivirus, and the transgene is stably expressed in the resulting cell line and is transmitted to daughter cells during cell division. Consequently, the absence of staining at later stages is indicative of the IMEF enzyme’s inability to produce MNPs in the absence of a source of divalent iron.

In view of the results obtained, cell samples were prepared for the MPQ analysis. The MPQ method has previously demonstrated its efficacy in detecting magnetic particles generated inside Qt nanocompartments (Gabashvili et al., 2023). In addition to the previously enumerated advantages of the MPQ technique, it should be noted that the proposed approach allows for the determination of magnetic signal magnitudes in both living cells and fixed tissue samples. Additionally, the MPQ method has been demonstrated to facilitate non-invasive real-time *in vivo* detection of MNPs - labeled cells in the bloodstream (Gabashvili et al., 2025). The primary constraint of the employed approach pertains to the capacity of the MPQ device, which is only capable of detecting magnetic signals in magnetic materials characterized by nonlinear magnetization. For instance, antiferromagnetic ferritin evades detection by MPQ due to its low magnetization and linearity in the magnetic fields employed (Zelepukin et al., 2021).

**FIGURE 4:**
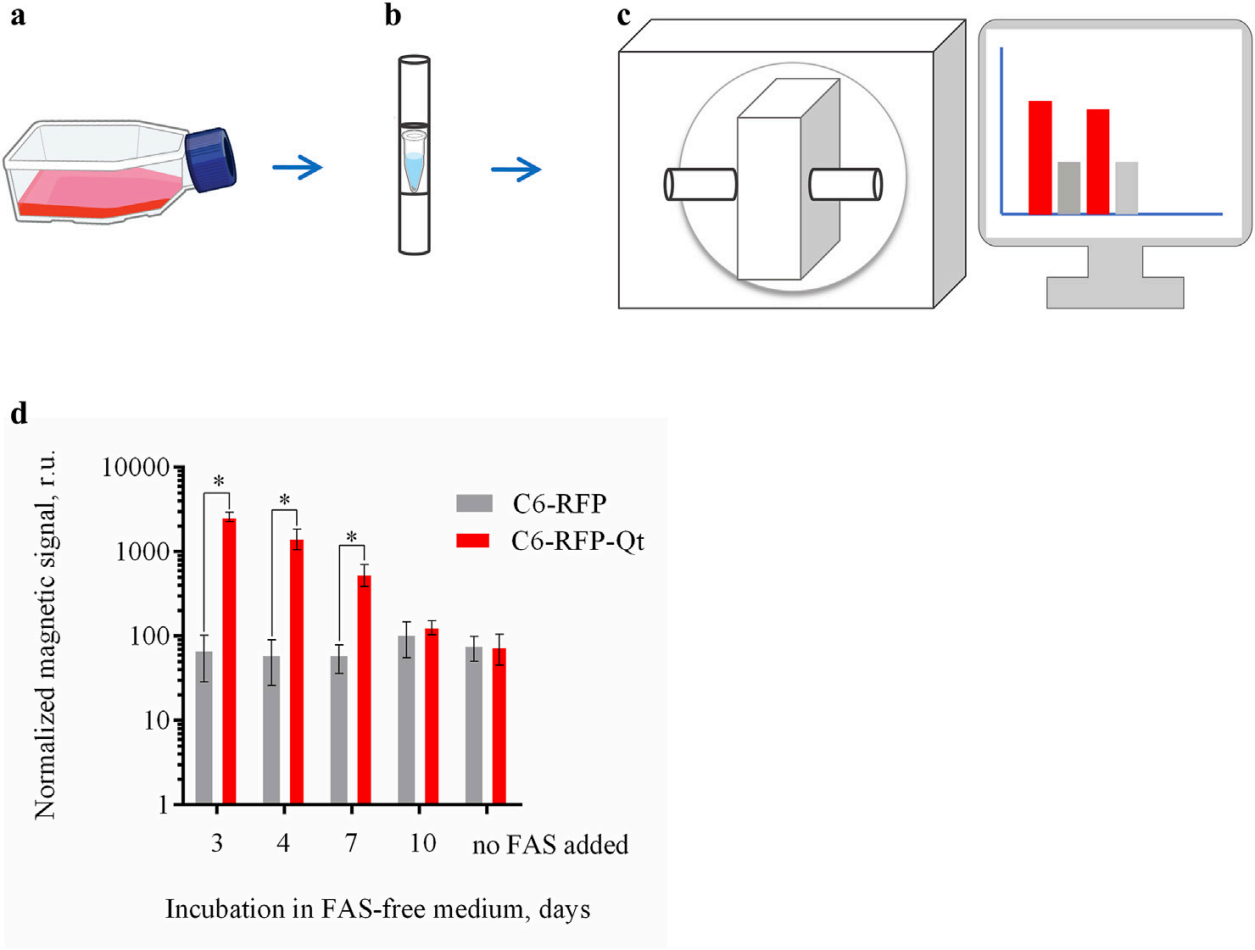
Dynamic evaluation of the magnitude of the normalized magnetic signals in C6-RFP-Qt and C6-RFP cell pellets using the MPQ method. **(a)** The cells were incubated with 4 mM FAS solution in cell culture media for 48 h; **(b)** the growth medium was substituted with a FAS-free medium, and the cell pellets of 1.8 × 10^6^ cells per sample were prepared at days 3, 4, 7 and 10; **(c)** cell pellets were placed in the MPQ detection coil; **(d)** magnetic signals registered by the MPQ device at days 3, 4, 7, and 10 after the culture medium replacement. The data are shown as the mean ± S.D. of three independent experiments, * indicate *p* < 0.0001. Sources: bioart.niaid.nih.gov/bioart/143; bioart.niaid.nih.gov/bioart/303.

Prior the analysis the cells (C6-RFP-Qt and C6-RFP) were incubated with FAS, followed by the exposition in a FAS-free medium (Figure 4a) as was previously described. The cell pellets were prepared at days 3, 4, 7 and 10 (Figure 4b). The cuvettes containing the cell pellets were positioned within the detection coil of the MPQ device (Figure 4c). The magnitude of the normalized magnetic signals was determined in cell pellet samples of 1.8×10^6^ cells per sample (Figure 4d).

The data obtained are in a good agreement with the results acquired when assessing the iron amount in the cells using the Perls staining. The maximum value of the normalized magnetic signal was recorded on day three, after which there was a gradual decrease in the signal. By day ten, the signal value in the C6-RFP-Qt cell samples was not statistically significantly different from the value in the control.

In the context of biotechnological applications, bacterial nanocompartments is a multifunctional tool. For example, these proteins have been utilized as nanobioreactors (Diaz et al., 2021; Kwon et al., 2024), delivery systems (Van de Steen et al., 2021; Gabashvili et al., 2022a; Gabashvili et al., 2024), as well as labels for transmission electron microscopy (Sigmund et al., 2019; Sigmund et al., 2023), and MRI (Gabashvili et al., 2021; Gabashvili et al., 2022b). Among the aforementioned approaches concerning the application of nanocompartments in the field of biotechnology, significant expectations were placed on the enhancement of presently available genetically encoded MR reporter systems. Nanocompartment-based MR reporters offer several advantages over other MR reporter genes commonly used for imaging mammalian cells. For instance, the expression of Divalent Metal Transporter 1 (DMT1) has been demonstrated to induce MRI contrast in various cell types, including glioma, both *in vitro* and *in vivo*. A disadvantage of this system is that MRI is only possible after systemic injection of MnCl_2_ (Bartelle et al., 2013). Another frequently used MR reporter is the transferrin receptor (TfR), which facilitates the internalization of transferrin-bound iron atoms into cells. However, despite the fact that TfR1 expression is generally well-tolerated by cells, achieving sufficient iron accumulation for a significant MR signal often requires the co-expression of ferritin. In this regard, both ferritin and transferrin are significantly inferior to encapsulin in terms of the number of deposited iron atoms (Naumova and Vande Velde, 2018).

Despite the capacity of encapsulin-based labels to facilitate the visualization of cells using MRI in SWI (susceptibility weighted imaging) and T2* weighted images, there is currently no method available for the *in vivo* loading of divalent iron into nanocompartments. At present, this tracking strategy is particularly well suited for such cells as MSCs, as well as for professional phagocytic cells, including macrophages, preincubated with divalent iron prior to engraftment. A strategy for employing encapsulins in the tracking of rapidly growing tumors has yet to be developed.

## 4 Conclusion

In this study, we generated a rat C6 glioma cell line with stable expression of two genetically encoded proteins–RFP and Qt nanocompartment. The cells were characterized by ICP-MS and Prussian blue staining. Quantitative assessment of iron accumulation in C6-RFP-Qt cells showed that after 4 mM FAS addition, iron accumulation in cells has a dose-dependent character. The amount of intracellular iron in C6-RFP-Qt cells is significantly higher compared to control C6-RFP cells. This feature enables the visualization of C6-RFP-Qt cells in the Prussian blue reaction. In the absence of FAS, the magnetic signal in C6-RFP-Qt remained discernible for 5 days.

## Data Availability

The original contributions presented in the study are included in the article/Supplementary Material, further inquiries can be directed to the corresponding author.
