# Supplementary material for: Detection of biogenic magnetic nanoparticles in rapidly dividing tumor cells by the nonlinear magnetization method
Source: Front Bioeng Biotechnol. 2025 Oct 27;13:1680057. doi: 10.3389/fbioe.2025.1680057 (PMC12598001; doi:10.3389/fbioe.2025.1680057)
Supplement: Supplementary file 1 [file Supplementaryfile1.docx]

Supplementary Material: Detection of biogenic magnetic nanoparticles in rapidly dividing tumor cells by the nonlinear magnetization method

Marina V. Milovanova, Anna N. Gabashvili, Elizaveta N. Mochalova, Ekaterina O. Gurtovaya, Irina E. Egorova, Anastasiia A. Dresviannikova, Olga Yu. Griaznova, Petr I. Nikitin


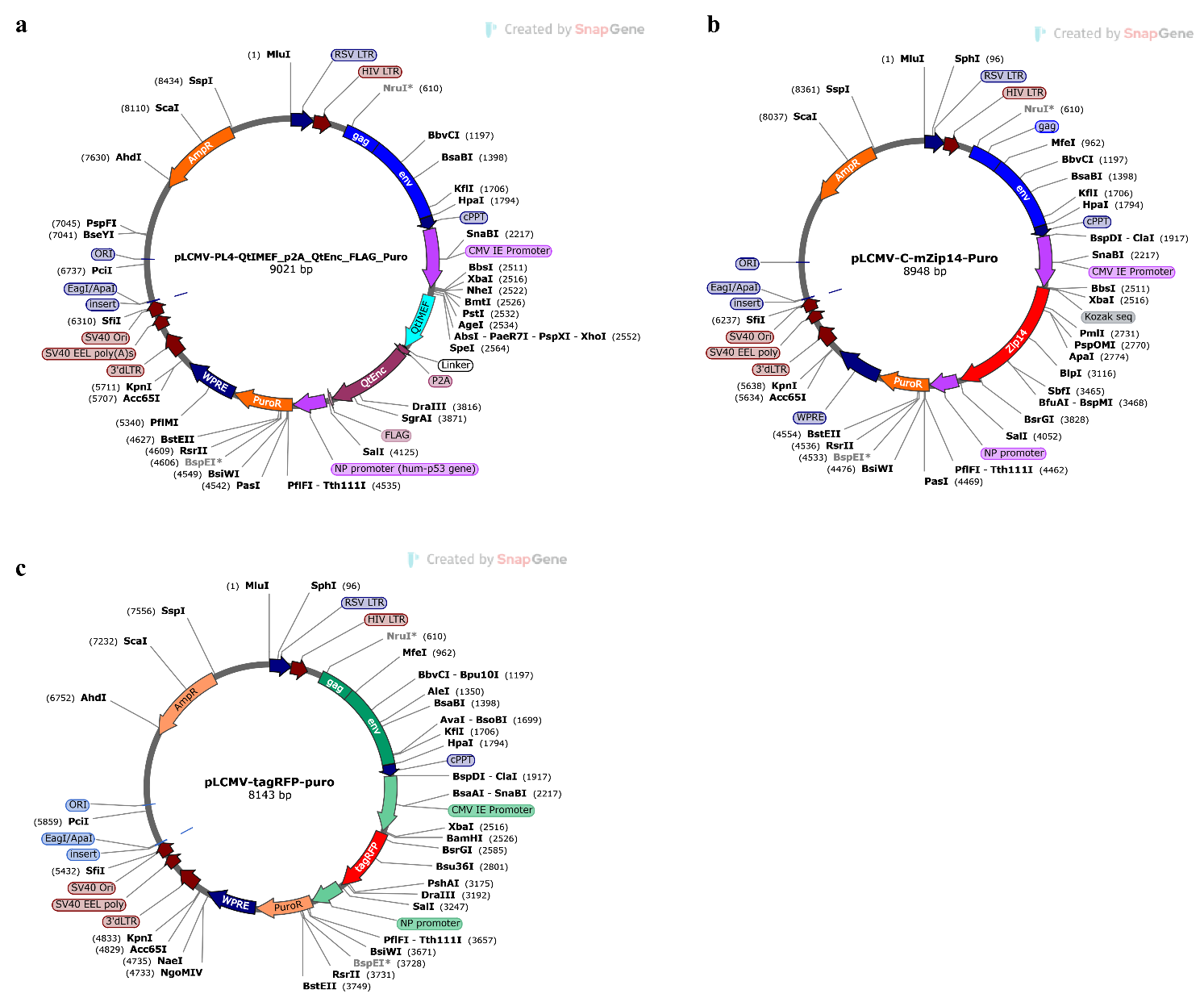


**Supplementary Figure 1.** Maps of plasmids carrying *Quasibacillus thermotolerans* nanocompartment (a), the divalent metal transporter mZip14 (b) and RFP (c) encoding genes.


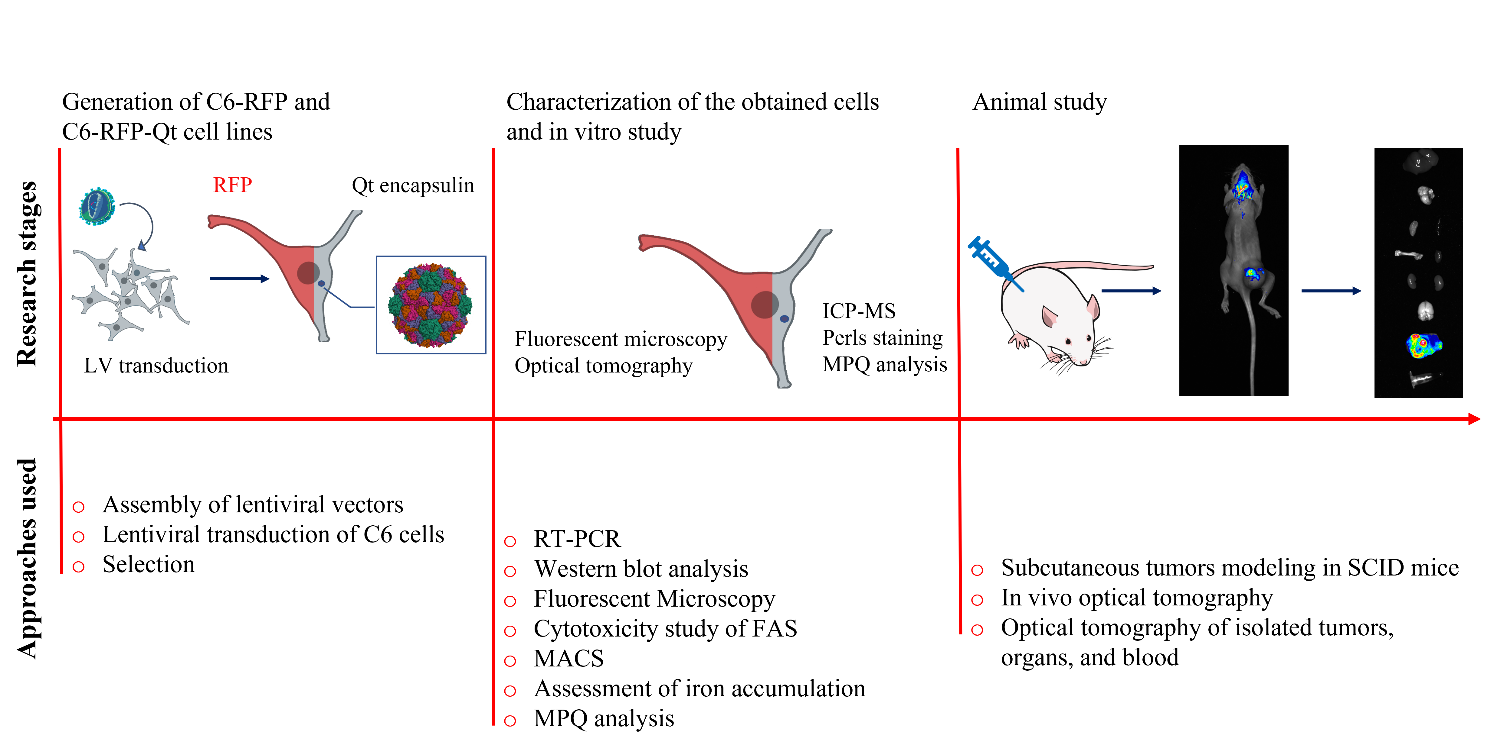


**Supplementary Figure 2.** A schematic summarizing the comprehensive workflow, from the generation of a transgenic cell line to animal studies. Sources bioart.niaid.nih.gov/bioart/614; bioart.niaid.nih.gov/bioart/198; bioart.niaid.nih.gov/bioart/283.
